# Lost in pandemic time: a phenomenological analysis of temporal disorientation during the Covid-19 crisis

**DOI:** 10.1007/s11097-022-09847-1

**Published:** 2022-09-02

**Authors:** Pablo Fernandez Velasco, Bastien Perroy, Umer Gurchani, Roberto Casati

**Affiliations:** 1 Trinity College Dublin, Dublin, Ireland; 2 Institut Jean Nicod (ENS, EHESS, CNRS), Paris, France

**Keywords:** Covid-19, Time experience, Temporal disruption, Phenomenology, Pandemic

## Abstract

People have experienced many forms of temporal disorientation during the Covid-19 crisis. For this study, we collected a rich corpus of reports on the multifaceted experiences of disorientation during the pandemic. In this paper, we study the resulting corpus using a descriptive approach. We identify six emerging themes: temporal rift; temporal vertigo; impoverished time; tunnel vision; spatial and social scaffolding of time; suspended time. We offer a phenomenological analysis of each of the themes. Based on the phenomenological analysis, we draw a key distinction between episodic and existential forms of temporal disorientation, and we argue that the Covid-19 crisis is best conceptualised as a period of suspended time.

## Introduction

The Covid-19 pandemic has deeply disrupted the landscape of our collective lives. Lockdowns, curfews, distancing measures and a generalised uncertainty have had a lasting psychological effect on the population (Pedrosa et al. 2020, Serafini et al. 2020, Pratti and Mancini 2021). The landmarks we use to orient ourselves in our everyday life have been shaken, and, as a result, many find themselves in a state of disorientation that has spatial, temporal, and social ramifications (van Gils-Schmidt et al., 2020; Nelson, 2020, Fernández Velasco et al., 2021). The concept of disorientation has been employed to further our understanding of the pandemic in work coming from philosophy, ethnography, geography, cultural studies and social psychology (Henk, 2020; Oinas, 2022, di Friedberg 2021, Means & Slater 2021, Benedikter & Fathi, 2021, respectively).

This concern with the disorienting nature of the pandemic is also reflected in the public realm outside of academic discourse, ranging from discussions in social media platforms to journalists writing about how disorienting “watching the world snap shut” was (Wetsman, 2020), about “New York’s dreamy, disorienting reopening” (Pillsbury, 2021), about the way in which data from new variants “remains obscured by a thick and disorienting fog” (Nuki, 2021), or more recently, about the Omicron variant and how it “ushered in a new and disorienting phase of the pandemic” (Bosman 2022). Overall, the notion of disorientation seems to capture in a meaningful way some of the chief underlying dynamics of the Covid-19 experience.

At the onset of the pandemic, we set out to study the Covid-19 crisis through the lens of disorientation. The present paper developed in the context of this larger enterprise. Our interdisciplinary team involves philosophers and data scientists, and our work ranges from the analysis of social networks to the evaluation of public health policies. Central to our work is the development of quantitative and qualitative questionnaires designed to explore the impact of the pandemic (Velasco et al., 2022). As a result of these efforts, we have collected a rich corpus of reports on the multifaceted experiences of disorientation during the Covid-19 crisis.

Analysing these reports can be done through tagging and coding, but that approach would omit some of the most philosophically and phenomenologically interesting aspects of the corpus. In this paper, we offer a complementary, descriptive approach. We undertake a phenomenological analysis to elucidate some emerging themes from the corpus pertaining specifically to temporal disorientation. Examples of emerging themes are the rift between pre- and post- pandemic perceived time, the sensation that time itself has been put on hold, or the temporal vertigo experienced due to the feeling that time passes at once faster and slower. Based on these emergent themes, we draw a key distinction between episodic and existential forms of temporal disorientation, and we argue that the Covid-19 crisis can be fruitfully conceptualised as a period of suspended time.

Following the present introduction, Sect. 2 offers a conceptualisation of the disorienting aspects of the pandemic experience. Section 3 outlines our research methodology. Section 4 explores the emerging themes from our collected corpus of reports. Section 5 discusses the broader implications of this thematic analysis for our understanding of the Covid-19 crisis.

## Disorientation and the pandemic

The recognition that there are non-spatial forms of disorientation certainly preceded the Covid-19 crisis. Researchers coming from a broad variety of fields had previously investigated social disorientation (Papadopoulos, 2002; Bissell & Gorman-Murray, 2019), cultural disorientation (Potosky, 2016), political disorientation (Tonello, 2018), temporal disorientation (Giannakopoulos et al., 2000), and the disorientation of illness (Lajoie, 2019) disability (Parrey, 2020) or cognitive inquiry (Earnshaw, 2019).

Of course, one must proceed with caution. One might worry that the above-mentioned authors use disorientation figuratively, without any strong theoretical implications. The reason for concern stems from two connected worries. The first is that spatial metaphors are widespread: we often talk of how we “feel down”, of the economy “going downhill”, or we describe how a friend was “in high spirits”. The second worry is that disorientation itself is a particularly effective figure of speech, which is attested by how pervasively the term appears in the literature (Alexander, 1996; Cresswell, 1996; Crang, 2001; Stiegler, 2009; Cadet, 2010; O’Neill, 2011; Marouan & Simmons, 2013; Saunders, 2016; Schmidt di Friedberg, 2017). Arguably, some of these uses will be intended only figuratively, and some of them will go further than that. The question is in what specific sense temporal, social, or political disorientation can be said to be like the paradigmatic spatial case.

In a recent contribution, Fernandez Velasco et al., (2021) argue that disorientation should be understood as the experience resulting from the evaluation and regulation of processes integrating frames of reference pertaining to a variety of domains. Central to this account is the conception of disorientation as a metacognitive feeling — an affective state that emerges as a result of the tracking of cognitive processes (for a review of metacognitive feelings, see Arango-Muñoz and Michalean, 2014).

The idea is that there are representations in temporal, social, and political domains that are framed in similar structures to spatial representations of a physical environment. The relevant relations are cast in allocentric frames of reference and then integrated with representations in egocentric frames of reference to guide action (Klatzky, 1998). Egocentric frames of reference are centred on the subject (e.g. the table is to my right). In contrast, allocentric frames of reference are world-centred or environment centred (e.g. the table is in front of the wardrobe). The two reference frames are integrated to guide orientation. When this process malfunctions, the metacognitive feeling of disorientation ensues, resulting in a phenomenology often characterised by anxiety, helplessness, and isolation. While most research tends to focus on the negative aspects of disorientation, it is worth noting that the phenomenon is sometimes associated with a positive valence, linked to a sense of liberation and discovery (Schmidt di Friedberg, 2017). What is key for the current discussion is that the same affective structure holds for temporal, social or political disorientation as for spatial disorientation, because a single system of representation extends across all these domains.

The notion that a single system of representation extends across spatial and non-spatial domains alike is supported by mounting evidence in neuroscience showing that the hippocampus –traditionally conceived as the seat of spatial cognition– maps also non-spatial dimensions (for a review, see Epstein et al., 2017). Most notably, the hippocampus has been shown to be involved in representing time (MacDonald et al., 2011) as well as social (Tavares et al., 2015), conceptual (Constantinescu et al., 2016) and semantic dimensions (Solomon et al., 2019; Viganò & Piazza, 2020) in a similar way to how it represents physical space.

With this conception in mind, we can take existing assertions about non-spatial forms of disorientation in a way that goes beyond a figurative use. Thus, we can understand political disorientation “as a phenomenon that has striking similarities with the physical disorientation created by an alien landscape” (Tonello, 2018: 114); cultural disorientation as the affective experience that ensues when subjects “lack interpretive frames during episodes of cross-cultural interaction” (Potosky, 2016: 228), and instances of social disorientation as feelings that “signal a loss of bodily capacity to know others; to know how to proceed; and to know how to hold a situation together” (Bissell & Gorman-Murray, 2019: 2). Overall, disorientation, in its many dimensions, becomes “what happens when the interpretative grids we use to make sense of the world change rapidly and/or radically” (Saunders, 2016: 92).

The above conceptual work paves the way to a more precise understanding of the effect of the pandemic, in which the disintegration of the frameworks that we habitually used for navigating our social, temporal or political world has resulted in a multifaceted and generalised state of disorientation:

The COVID-19 pandemic must be considered as an extreme life situation, not only because it presents a concrete, well-documented life-threatening risk, but also for how it introduces a set of fractures into our daily life experience … Such situations remove any protective layering we develop throughout our lives that stabilise us and allow us to pursue individual, professional, or social paths in life. This deconstruction of self-identity can be linked directly to *the breakdown of established frames of reference* through which we construct ourselves in a social context by conforming to societal norms, group expectations and social pressure.

-Tarquinio, 2020: 3; our emphasis.

In what follows, we will focus on the temporal domain and explore the temporal disorientation that has emerged from the pandemic conjuncture. Applying the above domain-neutral definition of disorientation to this case, we obtain an understanding of temporal disorientation as the metacognitive feeling that results from the evaluation and regulation of processes integrating temporal frames of reference at different temporal scales. Temporal orientation involves the dynamic mapping of events in terms of their boundaries, their order, or their relative distance from each other and from oneself. When this process deteriorates –as was common during the pandemic– temporal disorientation emerges.

## Methodology

The aim of our qualitative questionnaire was to inquire into the heterogeneity of experiences of disorientation that had resulted from the Covid-19 crisis. We were wary of priming subjects, so we made sure to phrase our questions in an indirect way. We included three distinct, open-ended questions. We were especially interested in exploring temporal, social, and epistemic disorientation. Accordingly, we asked participants to discuss, first, any time distortions they might have felt during the Covid-19 crisis; second, ways in which they might have felt socially out of place; and third, ways in which they might have felt confused while navigating the information related to the pandemic. Each of these included some examples to aid the participants in understanding the questions. The Pôle Éthique of the Institut des Sciences Biologiques (INSB) of the Centre national de la recherche scientifique (CNRS) waived all ethical approval for fully anonymous questionnaires. The study has been conducted according to the principles expressed in the Declaration of Helsinki. We distributed the questionnaire through the mailing lists of universities in France and the UK in March 2021. 161 participants completed at least one of the three questions. 98 participants answered the French questionnaire, and 63 answered the English questionnaire (see Table 1 for the characteristics of the sample).

**Table 1:** sample characteristics

Characteristics	Value
Number of respondents	149
Respondents of the French questionnaire	95 (64%)
Respondents of the English questionnaire	54 (36%)
Average age	37.9
Median age	33.5
Female respondents	102 (68%)
Male respondents	43 (29%)
Respondents located neither in France nor the UK	28 (19%)

The aim of the questionnaire was, on the one hand, to collect a corpus of reports for further analysis, and, on the other, to use the reports themselves to design a quantitative questionnaire. The development of this quantitative questionnaire is discussed elsewhere (Velasco et al., 2022). As for the analysis of the corpus itself, it can be undertaken through coding or through reflexive description. Each of these methods has its advantages and its blind spots. Here, we pursue a descriptive approach (for a coding approach to the same corpus, see Perroy et al., 2022), which can give us insight into philosophically and phenomenologically valuable elements of the corpus that an analysis based on coding would be unsuited to capture.

The responses to the social disorientation question formed a fairly homogeneous whole and therefore did not contain a rich variety of emerging themes. In contrast, the responses to the epistemic disorientation question lacked the necessary thematic unity, which might indicate that the question itself failed to target a coherent whole. However, the responses to the temporal disorientation lay on an ideal middle ground: they are unified enough to be interrelated, yet heterogeneous enough to contain multiple themes that can be clarified through analysis. Consequently, in this paper we will focus our analysis on the responses to the temporal disorientation question.

Our approach can be understood as a process of thematizing meaning, which is one of the few aspects that is shared by competing qualitative approaches (Holloway & Todres, 2003). One popular method to thematise meaning in qualitative psychological research is the aforementioned coding thematic analysis, in which codes develop into themes (Braun & Clarke, 2006). Another method originates from the hermeneutic phenomenological tradition and aims to illuminate the hidden meanings of lived experiences through interpretation (Ho et al., 2017; van Manen, 2016). Finally, there is a thematic analysis approach that is based on the tradition of descriptive phenomenology (Sundler et al., 2019). In Sect. 4, we will follow this latter descriptive approach to outline and clarify the themes generated from the corpus. In Sect. 5, we will advance some theoretical reflections based on that analysis.

Some researchers talk about themes in terms of search and discovery – of uncovering themes that lie like “diamonds scattered in the sand” (Braun and Clarke 2016, 740). Guest, Bunce and Johnson aptly term this process “thematic discovery” (2006: 66). Braun & Clarke (2006) are critical of this angle, because talk of themes “being “discovered” is a passive account of the process of analysis, and it denies the active role the researcher always plays in identifying patterns/themes, selecting which are of interest, and reporting them to the readers” (p. 80). There is no denying that the present effort does not consist in passive observation. We set out to study the impact of the pandemic through the lens of disorientation. Our theoretical outlook guided the overall research process, including the design of the questionnaire. Therefore, rather than “thematic discovery”, we would describe the process as one of “theme generation”. Nevertheless, we still refer at times to “emerging themes” because the process is one in which themes emerge out of our interaction with and exploration of the corpus, not one in which themes are generated ex nihilo.

In this “theme generation” we had to balance the guiding aim of our inquiry with the pre-theoretical attitude that is characteristic of phenomenology. To do so, we followed the methodological principles advanced by Sundler and colleagues (2019). When following existing methodologies, there is always a danger of falling into ‘methodolatry’ (Chamberlain, 2000) or ‘proceduralism’ (King and Brooks 2017). Our research aims and topic being to a certain extent idiosyncratic, we were wary of giving precedence to rigid procedures over theoretical reflexivity. Part of the reason of following Sundler and colleagues is that they offer flexible starting points rather than codified rules for analysis.

What they suggest is to guide the entire research process by three interrelated methodological principles: openness, questioning pre-understanding, and adopting a reflexive attitude. Openness consists in questioning the understanding of the data and being attentive to the expression of experiences (Dahlberg & Dahlberg, 2003). Questioning pre-understanding requires an awareness of the preconceptions that might influence the analysis. The emphasis here is not so much on bracketing as in recognising our prejudice regarding what we already think about a phenomenon (inspired by Gadamer 2004). Finally, in Husserlian terms, what Sundler and colleagues refer to as adopting a reflective attitude involves a shift away from the natural theoretical attitude and towards a self-reflective attitude towards the data (Dahlberg et al., 2008).

To adhere to these principles, we carried out a process of triangulation in the generation of themes, with two complementary researchers performing the analysis (for an example of triangulation in thematic analysis, see Cassol et al., 2018). Furthermore, in the spirit of openness, we made sure that our guiding theoretical background (outlined in Sect. 2) was as explicit as possible, which helped us question ourselves and each other during the analysis.

Here again, we followed Sundler and colleagues three step process, only adding the extra element of triangulation to increase reliability: (1) Reading; (2) Search for meanings; (3) Organizing themes.

First, the two researchers familiarised themselves with the data through open-minded reading. They each read the full corpus several times in its entirety without note-taking. Then, the researchers searched for meanings of lived experiences in the text, marking them and describing them briefly. From these meanings, themes began to emerge. Finally, findings were written down and edited, and themes were named and described. While the idea of conducting a phenomenological analysis preceded the collection of the corpus, the specifics of the process were fine-tuned to adapt to the particularities of the corpus.

Note that the aim of the present analysis is to elucidate the emergent themes, not to measure their frequency. As a result, there are some elements in the corpus (e.g., participants forgetting the day of the week) that repeat often but that did not make it into the emergent themes. The themes were selected for their estimated contribution to clarifying the temporal phenomenology of the pandemic.

The corpus is open source, so other researchers can of course tackle it from a different angle, and this might generate a complementary but slightly different set of themes. As we already mentioned, we have also followed an alternative coding-based analysis of the same corpus (Perroy et al., 2022), which, in contrast, does capture what we believe is an exhaustive list of codes tagging the text. However, it also side-lines meaningful but infrequent themes in the corpus. The raison d’être of the present analysis is partly to bring those meaningful themes into the light.

## Temporal disorientation: emerging themes

As a result of the analysis described above, we generated the following five themes: *temporal rift*; *temporal vertigo*; *impoverished time*; *tunnel vision*; *spatial and social scaffolding of time*. Additionally, we identified an overarching theme –*suspended time*–, which connects to the other five, and which helps us elucidate the way in which the explored themes converge.

In this section, we will go through each theme in turn, highlighting its chief characteristics and illustrating them with excerpts from the corpus. In the next section, we will connect these themes to the conceptual framework of disorientation. The excerpts of the corpus are indented. For those excerpts that were reported in French, the original has been translated into English (for the original French reports, see Anonymized, 2022).

### Temporal rift

Several participants reported a sense of the pandemic marking a before and an after, a temporal rift that separated the time of the pandemic from the time that preceded it.

Everything that happened before the pandemic feels like it happened in some distant era, in the ‘Before Times’.

Referring to the events that happened before the pandemic as belonging to the ‘Before Times’ denotes a marked difference between pandemic time and pre-pandemic time. In the excerpt above, the onset of the pandemic is even described as a change of era. For some people personally exposed at the onset of the pandemic, e.g. by falling ill or losing a relative without proper ways to grieve (Mortazavi et al., 2021), the temporal rift might have taken the form of a traumatic event, similar to the way one might conceptualise other traumatic episodes (such as 9/11; Seery 2008). For most, the precise day the temporal rift took place might be vague or indeterminate, making it unclear whether the rift is best characterized by a precise watershed. In all cases, the best way to conceptualize phenomenologically the rift is by appeal to the feeling that a large distance separates the subject from the events that happened before the pandemic, which we find consistently in our corpus.

I feel that everything that happened before the pandemic happened a long time ago.

This temporal distance is not just an illusory judgement. It also results in a different form of affective relation towards the period that lies beyond the rift:

The more this goes on, the more I have the impression that the pre-covid period belongs to an ancestral past recalled by oral tradition and a latent nostalgia rather than to a lived past and a living personal memory.

Here again, we see the notion that the time before the appearance of Covid-19 belongs to a different era (in this case, an ancestral past). This would make the pandemic itself epochal, i.e., an event that marks the beginning of a new period (Simon, 2020; Halmi, 2021), a notion deserving some caution. Whether the pandemic is epochal at the societal level is only for future historians to tell and outside the scope of this paper. The participants were reporting their experiences during the pandemic. It was certainly not an event in their past. In this respect, somebody in 2021 describing the pandemic as epochal is different from somebody in 2021 describing the fall of the Berlin Wall as epochal (e.g., Genz et al., 2021).

The ancestral past of the Before-times in the reports is put in contrast to a *lived past*. The rift does not just make some events appear as further away. In some cases, it makes those events appear as further away from oneself, and therefore, as not truly having been lived by the subject. The rift renders the memory of past pre-pandemic events impersonal, with an impoverished sense of mineness in comparison to memories from within the pandemic (for a discussion of memory and mineness, see Roache 2016).

Concluding from the corpus that there is a wide-spread impoverished mineness concerning pre-pandemic memories would be a somewhat extreme conclusion. What the description above points to, however, is that while distance is an important element characterising the current theme, there is more to the experience of a temporal rift than just distance. When participants indicate that the time before the pandemic feels like a different age or some ancestral era, they are not only talking about perceived temporal distance. They are also noting that the time before the pandemic feels different from the time during the pandemic. The events at the other side of the temporal rift are distant *affectively* as well.

Everyday decisions during the crisis were accompanied by a series of variables that were mostly absent before the pandemic, e.g. whether one should come at a party because of health concerns, or whether one should move to a different place more suitable for remote working. Something that might make the temporal rift be perceived as epochal are the ways in which previous everyday frames of reference and their subsequent actions are no longer relevant after the rift. This induces the affective distance with events at the other side of the temporal rift.

In psychopathology, a temporal rift is a hallmark of the switch from a pre-reflexive (or implicit) to a reflexive (or explicit) experience of time in instances of shocks or sudden unexpected events (Fuchs, 2013). A rift such as the one induced by the Covid-19 pandemic typically “breaks through the habitual” (p.79) and disturbs the “affective-conative momentum” (p.78) proper to a pre-reflexive experience of time. As Fuchs puts it, “in such moments, pure lived temporality sustains a rift: “’now’ and ‘no longer’ are disconnected and create an elemental segmentation of time. What hitherto had been a timeless continuum splits off from the present and now turns into a remembered (and no longer a merely ‘retained’) past.” (p.79).

An interesting upshot of this difference between the time before and during the pandemic is that ‘to many people’ the time of the pandemic feels longer than it should:

I feel like the pandemic time (that year) has an outsized position in my mental timeline. It’s felt like an impossibly long year, and when I reflect back on it, it feel as if it takes up much more of my life than other recent years.

Another participant reports:

One year has passed since March 2020 and it feels like 5.

All in all, the onset of the pandemic was lived as temporal rift akin to a change of era, as demonstrated phenomenologically by the many reports emphasizing the distance between oneself and events before the rift. This distance has clear affective implications, which is shown by some memories before the rift being described as impersonal, or no longer relevant to one’s situation, as well as feeling the times since the pandemic began occupying an outsized proportion in one’s mind compared to what’s over the rift. This affective distance indicates the temporal experience during the pandemic is as disrupted as previous times feel far away.

### Temporal vertigo

Numerous reports testify contradictory feelings such as time passing both slower and faster, or both shorter and longer. These contradictory experiences, lived at once, are in themselves disorienting: only the (egocentric) feeling of subjective temporal distances as well as the (egocentric) feeling of passage of time are distorted, rather than the beliefs about objective time duration. This egocentric disorientation is thus perspectival and based on conflicting feelings. It is best characterized as a form of temporal vertigo, akin to the vertigo induced while reflecting on the discontinuities and continuities of oneself while ageing (Segal, 2014).

The pandemic itself seems to be going on for both ten years, and two weeks. It’s difficult to conceptualise and uncanny.

The previous report is about time being long and short at once. The following one, an even more frequent phenomenon, is about time passing both faster and slower.

It’s a rather curious period because it gives the impression of “a long time that passes very quickly”, it’s quite difficult to explain. Government measures such as curfews and the closure of leisure facilities (bars, cinemas, museums, etc.) result in a slowing down of social life and make the time feel long, but on the other hand, I see the weeks going by at full speed.

This participant describes a long everyday time. The days feel longer than normal. In contrast, the weeks pass quickly. As we shall see, two key elements to clarify this structure are temporal perspective and temporal scale. Temporal perspective has to do with where we are positioned with respect to the time that is the object of our description (e.g., feeling the present time flow vs. looking back at past time). The following report directs our attention to temporal perspective with a poignant metaphor:

I felt that time during the pandemic passed in the same way it passes during a very long trip in a bus: while it lasts, it feels eternal, as if you were always in that bus, but once it’s over (maybe even the minute you get down) it seems as if it never happened, as if an eternity was compressed in one second.

While time is passing, it feels slow, and it feels long. And yet, when one looks back at time past, it feels short and compressed.

It is the slowness of the repetition of a daily routine of remote work that strikes me the most. Time passes slowly. But paradoxically, once the weeks have passed, looking back, it seems to me that everything has passed very quickly, without anything notable. A life of details...

The participant attributes the slow flow of time to a monotonous routine, and time having passed fast to the lack of notable events. This is something that we find also in other reports:

Time seems to pass slowly and quickly at once: every day is the same, which creates boredom, at the same time there are no real events to anchor the memory to, which gives the impression that time passes quickly.

And again:

Time seems to pass both more slowly, since there is nothing to punctuate the infinitely continuous daily flow of the student in front of the computer, and more quickly, since the absence of any memorable past event provides no temporal landmark in the retrospective medium term.

Particularly surprising is that it is the lack of memorable events that makes time both flow slowly and pass quickly. Relatedly, the same difference between fast and slow time can be conceptualised as two speeds of time at two respective temporal scales:

I feel that individual days are dragging or feel longer than usual, but months are passing very quickly.

Notice how the experience above is framed using the same perspective for the (slow) passage of days and the (fast) passage of months. The same is true in this other report:

The weeks are passing incredibly quickly. I cannot believe that a year has gone by since this all started … However, each day feels like it lasts forever, and I cannot wait for night so I can get into bed.

It seems then that some participants make sense of the contradiction by appeal to temporal perspective and some by appeal to temporal scale. One could interpret this as different subjects talking about different types of experience. We would rather interpret the explanations as complementary, as two sides of the same coin. The shorter scale is associated with slow time. The longer scale is associated with long time. Accordingly, the flow of time (on a day-to-day basis) is slow, while the time that has passed (looking at longer timeframes) seems to have passed quickly. Participants appeal to either perspective or scale because they only need one of the explanatory elements to communicate their experience.

Nevertheless, the fact that there are two ways of making sense of the tension does not make that tension vanish. It goes back and forth from one temporal horizon to the next, and it makes the temporality of the pandemic hard to grasp:

This takes the form of both longer and shorter time, punctuated only by fixed things that are ultimately few in number, such as meals and bedtime. In between, time seems elastic and very undefined.

In the end, the varying speed of passage of time and the varying felt distances, which depend on the temporal scale and the temporal perspective behind the horizon of experience, made people experience temporal vertigo. Such reflexive vertigo is disorienting insofar as it makes it hard for people to realize in their mind the current temporal layout. As we shall now see, the vertigo was experienced on an impoverished temporal plane.

### Impoverished time

The monotony of everyday life during the pandemic has, for many participants, meant not only alterations in the perception of time, but an impoverishment of the very texture of time:

Time moves very quickly. The patterns of the day (eating, cleaning, chores, preparations) expand from being mere patterns to being the whole of time.

The report above talks of a time that has been reduced to the “patterns of the day” – the monotonous routine that, as we just saw, also contributes to both the slowing and the quickening of time. The pre-pandemic time had more to it than routine activities. There were events that punctured the routine and changed the rhythm of life and with it the quality and texture of time itself. During the pandemic, it is this texture that has been sanded down. What we are left with is a flat, impoverished time:

I have the feeling that time has passed ‘strangely’, generally speaking, in the sense that all this was not planned, and especially in the sense that daily life is more ‘flat’ than usual.

Another participant links this explicitly to the lack of anticipated events that broke the routine (in this case, holidays):

Suddenly the year that was punctuated by this regenerative interruption was completely tarnished and lost its meaning. Meaning is made precisely by escaping, by taking the necessary steps back to break up a daily routine that seems, without that, to be a day without end.

The year of the pandemic has become tarnished, dull, without events to hinge upon. The result is a day without end, a Groundhog Day. This other report points in a similar direction:

The days seem to repeat themselves. In my weeks, there are no more events, only non-events.

This concept, non-event, is rather perspicacious. Events occupy differentiated regions of time. A day that repeats itself seems to be an undifferentiated region of time. Within it there are only non-events. We are left with a time that is flat, a time that is empty of authentic events. The experience of such an impoverished time –depleted of everything but the patterns of the day– brings with it an awareness of time’s fragility:

Like an awareness of the fragility of time: the two lockdowns experienced in slow motion, the summer accelerated in a lightning fashion, the screen time, usually sporadic, which now makes up most of my days ... Everything seems elastic and mouldable, there are no more stable landmarks, and what appears is a kind of paranoia.

This fragility translates into the previously explored temporal vertigo being subjugated to time itself. Claims of longer and shorter temporal distances end up escalating to claims of time dilating or contracting.

I certainly felt both a time dilation and a time contraction: time is a spring.

In these instances, it is as if time itself, rather than the individual’s perception of it, was distorted.

Time definitely passes more slowly -- it seems like the past year has been one very long weekend.

All in all, the time of the pandemic appeared as devoid of punctuation, anticipation, and salient boundaries, which made its texture sanded down. It is a temporal landscape without temporal landmarks. So sanded down, in fact, that people could attribute their vertigo to time itself under the form of contraction or dilation. What we’re now going to see, is that the impoverished sense of time during the pandemic, as well as the previously seen temporal rift, both contributed to people experiencing tunnel vision with respect to their future.

### Tunnel vision

I often have the feeling of being in a tunnel, without seeing the exit.

We started out thematising a temporal rift between pre-pandemic and pandemic time. We showed how the pre-pandemic time feels distant, even impersonal at times. We showed as well how ‘in the pandemic’ time is experienced as flowing slowly and yet, once past, it seems to have passed at high speed. But what of the time that lies ahead? What of post-pandemic time? In many reports, we witness the difficulty people find when it comes to projecting themselves into the future, specially so to a time after the pandemic. Following the report above, we call this *tunnel vision* — the sensation of looking at the future and not being able to imagine anything different than monotonous repetition:

The future seems absolutely inaccessible, and the impression of being stuck in an unpleasant perpetual present where nothing shall ever happen is tenacious.

At the heart of this phenomenon is an abnormal difficulty in projecting oneself into the future. A difficulty that can in itself be crippling:

I feel unable to project myself and I easily fall into a spiral of emptiness where I cannot do anything productive for several days. I feel very sluggish and unmotivated.

*Tunnel vision* leaves people unable to go on. They cannot imagine future states of affairs, and this difficulty in looking ahead means that one is adrift, unable to orient oneself forward in time:

I lost a sense of time, of urgency, of what needs to be done and accomplished when.

The difficulty lies on the one hand, in the lack of motivation that comes with losing one’s sense of a future stretching ahead. On the other hand, the difficulty is also that people, by necessity, have to make decisions about the future even if that future seems inaccessible:

Moreover, it is very difficult for me to project myself into a medium or long-term future. Nevertheless, despite these ‘blinkers’, I still make decisions that have a long-term impact, such as moving house, entering a civil service examinations, etc.

We noted in the beginning of this section that the *temporal rift* separating pre-pandemic and pandemic time often resulted in pandemic time taking an outsized dimension in people’s mental timeline. When it comes to *tunnel vision*, we see that the outsized time of the pandemic stretches unnaturally not only into the past but also onwards into the future:

Because my own government has fumbled their vaccine efforts so badly, and there’s little hope of mass vaccinations here before the summer, the next six months or so also loom large in my mind, feeling as if they stretch out forever.

In the end, the difficulty to affectively relate to times before the pandemic, as well as the difficulty to anchor landmarks on an impoverished pandemic time, due partly to vertigo, contribute to not feeling at ease with future landmarks and horizons. The distortion of agency in the present, with feelings of being stuck, crippling lack of motivation, and future-oriented anxiety and hopelessness, all contribute to people experiencing a sustained form of tunnel vision. To better characterize the overall temporal experience during the crisis, we are now going to address the interaction between time on the one hand, and spatial and social features of time on the other. This will lead us to claim that the overall temporal experience during the Covid-19 crisis could be best described as suspended.

### The spatial and social scaffolding of time

We have discussed the effect of monotony and of the lack of landmark events on the temporality of the pandemic but we didn’t investigate yet its interplay with the social and spatial dimensions. The notion of scaffolds points to the various properties of the cognitive or affective attunement of the mind with one’s surrounding environment, and to what extent the latter exercises a causal relationship on – or is constitutive of – the former (Sterelny, 2010; Saarinen, 2020). Accordingly, an aspect that is lurking in the background and that many participants reference explicitly is the way in which various social configurations structure temporal experience:

Without social “obligations” (obligations also in a positive sense) life was easier for a while. However, I also felt that structures started to get lost (when do I get up? when do I go to bed? when do I work? when do I not work? what will I do when I am not working?).

The rhythm of everyday life is often constrained by social demands and conventions. There is a time at which one needs to go to work and a time at which work ends. And because there is a time one needs to go to work there is a time at which one should wake up and by extension a time at which one should go to bed. These are the kind of scaffolds that disintegrated during the pandemic for many people. The subject above loses the said scaffolds and does not know how to proceed. Moreover, time is not only scaffolded socially but also spatially:

Loss of temporal reference points which are not only linked to the suspension of time but also, I think, to the deprivation of space. Not being able to change space.

Here, the subject feels disoriented (loses the relevant temporal reference points) not only because of the suspension of time, which based on the analysis above we can characterise in terms of the lack of events and the associated monotony. It is also a spatial affair. Not being able to leave the house, to change place, results in an impediment to locate oneself in time. Time is not only scaffolded via social structures but also via activities that are spatially extended. When we are not home bound, we don’t just start work, we *go* to work. This spatial scaffolding of time aligns with mounting empirical evidence that movement transforms the perception of time (De Kock et al., 2021).

Lived time is scaffolded socially and spatially, as seen by a growing number of studies that argue that memory and imagination are spatially scaffolded in ways essential for their experiential qualities and their organization (Robin, 2018). It is precisely the social and spatial scaffolds of lived time that came apart as a result of the Covid-19 crisis. Nevertheless, we shouldn’t consider subjects as passive casualties of the resulting disorientation. Many subjects report active strategies of disorientation remediation:

I know we often talk about the routine of everyday life, but usually it is imposed on us by schedules, it is the consequence of certain social imperatives. During the lockdown, I set it for myself voluntarily, and I think it was probably a way to have some control over the events, without getting completely caught up in this elongated time. I wasn’t afraid of not knowing the end date (of lockdown), I think, because we get used to not being able to control time in a precise way, we shift our attention to small temporal scales (a day for example).

The participant sets out to establish a new routine because the old one, established by external social imperatives, has fallen apart. This is explicitly described as a strategy to avoid succumbing to the feeling of elongated time of the pandemic (as we saw, the slowness of the flow of time comes from monotony at a daily scale). As such, the new routine is an affective scaffold insofar as the respondent trusts her new temporal structure to modulate her affective state– as seen in her hesitant recollection (Colombetti & Krueger, 2015).

Furthermore, there is a shift towards smaller scale as a way of coping with the pandemic. Overall, temporal disorientation seems to involve difficulty in dealing with the macro time scale, difficulty in dealing with the local time scale, and difficulty of coordinating macro and local. Consequently, people try to engineer their activities so as to overcome all these types of difficulty. In other words, temporal horizons themselves are transformed as a result of spatial and social scaffolding. This is something that we also find (with some chagrin on the part of the subject) in the following report:

Time is constantly dilating. Some days go by at breakneck speed and others are incredibly slow, so that the very notion of a “day” no longer means much. Personally, I count and measure time in half-days, if not in hours. I cannot project myself into the future, since I do not even know what I am going to do for the next thirty hours.

In many cases, this shift of attention towards shorter time scales is also ensured through activities that serve to mould the experience of time:

Gardening has become instrumental in marking the passage of time (e.g., watching plants bloom, small daily changes).

Gardening here serves at least two time-related purposes. Firstly, it makes one focus on “small daily changes”, which shifts attention to smaller timescales. Secondly, it helps one mark the passage of time, because there are subtle seasonal changes that one gets attuned to through gardening. This relates to another way of tackling the disorienting character of pandemic time, which is by focusing on the rhythm that is set not by social structures but by natural events:

All the months seem to be swallowed up into an undifferentiated whole because of the absence of birthday parties, family gatherings, holiday trips, etc. The only temporal rhythm that’s still to be experienced is the one by nature (the seasons, cold days being alternated by warm days, etc.)

Of course, temporal disorientation does not always lead to hearty activities like gardening. For many, being knocked off a habitual temporality results in more pernicious ways of adjustment:

The days feel far too short, so I end up staying up, sometimes as late as 8 am, to try to fit more into the day.

All in all, social and spatial features of everyday activities scaffold time. The very temporal horizon of experience changes as a result of this scaffolding. One could counter the many instances of disorientation we reviewed with temporal vertigo, impoverished time, or tunnel vision by affectively scaffolding a new time upon activities that prevented one from ruminating the past or having one’s glaze irresistibly focused on looming anxious possibilities. Nevertheless, even such a strategy can ultimately lead to time feeling suspended.

### Suspended time

Throughout the reports, many participants relate the lack of events during the pandemic to a sense of life being put on hold. We call this sense of time standing still *suspended time*. In our analysis, *suspended time* is an overarching theme, because (as we shall see in this subsection), it connects to the other five themes in a way that clarifies how they all hang together.

Here is how one participant describes *suspended time*:

It felt like time stood still for many months while life was ‘on hold’ and every day was similar, with no punctuation by landmark events.

Another participant describes time stopping:

I sometimes feel like that time has stopped, it’s like we skipped one year.

And here is one participant that describes living in a parenthesis out of time, because the lack of unexpected events means that there are no temporal landmarks:

I have weeks without a weekend, or even without a night..., I type and manage my diary to programme activities in video chat. No breathing, no breaks, no unexpected events... in these conditions I no longer have any temporal landmarks. I feel like I’m living in a parenthesis out of time.

When one is living in this parenthesis out of time, the activities themselves feel directionless, also in suspension:

Because work is not part of time, it doesn’t seem to get done, because it seems like it has no purpose and remains in suspension. In the meantime, the patterns of the day are necessary but monstrous … Cleaning the house, making yoghurt, showering. Didn’t I just do that? How is it possible I am doing this again?

As we mentioned, the themes generated during our analysis are all interrelated. Our taxonomy is flexible, and with permeable boundaries. It is not as if the experience of one participant corresponded to only one theme, another participant’s experience only to a different theme, and so on (although of course they might report only a slice of their broader experience). Monotony and the lack of landmark events resurface when discussing *suspended time*:

I had this strange feeling that time had stopped but I knew it was still passing and now I won’t be able to tell you what happened at the end of March, April or May last year because there are no highlights that allow me to situate myself during this period.

Because at once time stopped and kept going, the subject is unable to orient within that period. In it, there are no highlights, and once more, no highlights means no landmarks for temporal orientation. In the report below, 2020 is even described as a blank, an empty period:

I often think of 2019 as “last year”, 2020 seems not to have existed, like a blank, passed very quickly, without having left any marks.

Some describe the past year as non-existent. For some, the strange temporality of the pandemic can sway them into a sense of unreality, so that looking back imbues the past with an oneiric texture:

The past seems unreal, like a dream.

In some cases, *suspended time* is described in a very negative light. It is a black hole, a great void in which nothing matters:

The days seemed endless, the boredom was overwhelming me, I couldn’t work anymore, couldn’t function... what day is it? What time is it? What should I do? I could not answer these questions. This period seems to me to be like a big void, a moment when nothing mattered anymore, I was in a black hole during these few months.

Note that finding oneself in this big void means that the subject cannot answer what are at heart orientational questions. The subject has difficulty locating themselves in time (“what time is it?”), and this means that they have trouble deciding how to go on (“what should I do now?”). The disorientation and the indecision that come with *suspended time* leaves some people feeling that time itself is leaving them behind.

Loss of reference points between an impression of fixity (the days are very similar, they don’t seem to move) and the awareness that time passes and even that it passes quickly (already a year since the first lockdown): I have the impression that time moves on without me.

We have seen *suspended time* described as a great void and as a black hole, which are negatively connotated terms. However, just like with spatial disorientation (Schmidt di Friedberg, 2017), there are also some positive readings when it comes to the temporal disorientation of *suspended time*:

Last year until September 2020, I felt almost reassured with “time standing still”, I was not obliged to start my fieldwork at any cost, because I had a good excuse. The time seemed long, exiting lockdown reminded me of my adolescence, the carefree age where you don’t watch time go by, where you chat, hang out with your friends, go to your favourite activities...

If the conditions are propitious, being unable to go on also means a release from an obligation to go on. Notice how the description of a “carefree” time above resonates with the following description of the positive aspects of spatial disorientation: “Wandering around at random, almost purposely losing track, travelling around, trying to forget where you come from, where you are and where you are heading … Moving somewhere else not from obligation but for pleasure” (Baur in Schmidt di Friedberg 2017, p. 9).

Whether *suspended time* is experienced as a positive or as a negative occurrence might also change as the situation itself is prolonged. Below is an example of a participant that first tried the carefree approach but soon ended up feeling the already-mentioned sensation that time was slipping away:

I have felt stuck, as if my life could not progress in any direction. At first, I used it to try and relax and just be, but soon enough it felt like the standstill was destroying my ability to build a future, and I felt time was slipping away without me being able to do anything.

To conclude this overarching theme, it is helpful to consider the practical effects of experiencing *suspended time*. Just because subjects are able to reflect and to understand that time is not literally standing still (hence their description of time both flowing and standing still) we should not conclude that they are able to overcome the sense of time having stopped when it comes to making decisions. We have seen that many found it exceedingly difficult to work, or to function normally. To go even further, there is another report in which a subject describes how they broke up with their boyfriend before the lockdown, but they continued to see each other because the lockdown was a parenthesis in time, so that their being together did not count. In other words, some subjects took major life decisions based on the feeling that time hung suspended:

I separated from my boyfriend in February 2020, immediately afterwards we went into lockdown. We continued to see each other thinking that it was a “time out of time” that didn’t matter anyway. Since this summer, we have stopped seeing each other but the situation has not really changed, so I feel like the separation is very recent.

This notion of “time out of time” encapsulates well the central feature of temporal experience during Covid-19: the temporal rift from the previous mode of temporality made people experience vertigo to the point that time itself felt impoverished, and people felt stuck in a present without any future in sight. Even in those cases in which people found ways to scaffold time socially and spatially so as to resist this disrupted temporality, they still found themselves inhabiting a *suspended time*.

## Existential disorientation and suspended time

In the course of our thematic analysis, we have seen how the onset of the Covid-19 crisis created a temporal rupture –a before and an after– so that people found themselves dwelling in pandemic time. This rift resulted in different affective attitudes towards events lying inside or outside the pandemic, with past events feeling far away and often hard to relate to, and with future events appearing as out of reach and hard to project oneself into. Within the pandemic, people inhabit an impoverished, *suspended time*. In this temporal parenthesis, people reported a contradictory sense of the flow of time being unnaturally slow and of past events having passed quickly, even immobile. Key to understanding the peculiar temporality of this crisis is the breakdown of the social and spatial organisation that habitually scaffold lived time. Without these scaffolds, people become disoriented. Instead of landmark events punctuating the routine and providing anchors for orientation, during the pandemic, a widespread sense of monotony made for a day that repeated itself in perpetuity, as if time itself had been put on hold.

Commenting on the disorientation of the Covid-19 crisis, Matthew Ratcliffe advances a distinction between a limited experience of disorientation and an existential form of disorientation (2021). The former is an experience of uncertainty as to how to proceed. It is contingent and escapable. One only needs to reorient, and there is generally a context of social relations that can provide help. In contrast, the latter form of disorientation involves an inability to depend on others, a loss of the taken-for-granted habituality that once guided action. Ratcliffe makes the related remark that structures of orientation are often not only internalised frames of reference but a broader set of social relations on which one can rely for navigation. The issue with the pandemic, with its associated restrictions, is that it disrupts these interpersonal relations that would otherwise help us orient through uncertainty, resulting in a deeper, existential form of disorientation.

We had characterised temporal disorientation earlier on as the metacognitive feeling that results from the evaluation and regulation of processes integrating temporal frames of reference. If one were to put the emphasis on the frames of reference, it would seem that we are side-lining the broader orientational configurations that Ratcliffe brings to the fore. In contrast, we put the emphasis on processes. The Covid-19 crisis is also a crisis of the processes by which we orient ourselves spatially, socially, epistemically, and, in the case at hand, temporally. These processes themselves are widely distributed and they involve a complex social and spatial structure in order to function. When that structure crumbles, the orientational processes malfunction, and disorientation emerges.

There are three key limitations to the present study. The first one is that the work is on an exploratory, descriptive nature. A second, related limitation is that we had to phrase our questions to elicit rich responses without priming the respondent. While we believe we reached a very good balance in this respect, priming remains a risk to be acknowledged. Further work could operationalise the phenomenological claims emerging from our analysis into testable hypothesis to be tested through different methods. The second limitation pertains to the characteristics of our sample. Although we had over a hundred respondents, the large majority of them were in France or the UK. While the average age of 37.9 might be representative of the UK and France, the median age in our sample is only 33.5, and two thirds of our sample are female. Moreover, we distributed our questionnaire through university email lists, which means that our sample will be only represent a particular sector of the broader population. As a result, one should be careful when extending the conclusion from the current analysis to the general population.

Through our analysis, we further clarify the phenomenological structure of existential disorientation, focusing on the temporal case. The temporal disorientation that resurfaces throughout the discussion of our different themes is of a sustained, existential form. And the orientational structures that have come apart in the pandemic are not only internalised frames of reference. It is not even just about the inability to find others who can guide us through this uncertain landscape either. What we find is that the very spatial and social structures that scaffold our temporal orientation have come apart. As a result, we find ourselves lost in a wasteland of non-events, adrift in the suspended time of the pandemic.
